# Epigenetic Regulators in the Development, Maintenance, and Therapeutic Targeting of Acute Myeloid Leukemia

**DOI:** 10.3389/fonc.2018.00041

**Published:** 2018-02-23

**Authors:** Younguk Sun, Bo-Rui Chen, Aniruddha Deshpande

**Affiliations:** ^1^Tumor Initiation and Maintenance Program, Sanford Burnham Prebys Medical Discovery Institute, La Jolla, CA, United States

**Keywords:** acute myeloid leukemia, epigenetic therapy, leukemia stem cell, epigenome, chromatin modification

## Abstract

The importance of epigenetic dysregulation to acute myeloid leukemia (AML) pathophysiology has become increasingly apparent in recent years. Epigenetic regulators, including readers, writers, and erasers, are recurrently dysregulated by way of chromosomal translocations, somatic mutations, or genomic amplification in AML and many of these alterations are directly implicated in AML pathogenesis. Mutations in epigenetic regulators are often discovered in founder clones and persist after therapy, indicating that they may contribute to a premalignant state poised for the acquisition of cooperating mutations and frank malignancy. Apart from the proto-oncogenic impact of these mutations, the AML epigenome is also shaped by other epigenetic factors that are not mutated but co-opted by AML oncogenes, presenting with actionable vulnerabilities in this disease. Targeting the AML epigenome might also be important for eradicating AML leukemia stem cells, which can be critical for disease maintenance and resistance to therapy. In this review, we describe the importance of epigenetic regulators in AML. We also summarize evidence implicating specific epigenetic regulators in AML pathobiology and discuss emerging epigenome-based therapies for the treatment of AML in the clinic.

## Introduction

Acute myeloid leukemia (AML) is a clonal malignancy resulting from the transformation of hematopoietic stem and progenitor cells. AML is marked by enhanced proliferation and impaired differentiation of immature myeloid progenitors. Over the past few decades, strategies for treating AML have remained largely unchanged, although survival outcomes have improved, especially in younger patients (1). Despite these improvements, approximately 60% of young patients with AML eventually succumb to disease even after treatment with intensive therapies (2). In patients over 60 years of age, a population that has an increased frequency of AML, survival outcomes are much more dismal; less than 5% of patients are alive 5 years after diagnosis (3). There are several reasons why AML cure rates have plateaued. First, therapeutic approaches that have shown success in younger patients are often extremely aggressive and are, therefore, not tolerated well by elderly patients with frailty and other comorbidities. Treatment-related toxicity also results from the fact that standard therapies do not discriminate between normal and leukemic cells, resulting in severe toxicities. Second, although patient selection based on morphologic and cytogenetic features is routinely used for guiding treatment strategies and risk stratification, current therapeutic approaches do not adequately address the inherent molecular heterogeneity of AML. Last, current treatments that target the leukemic bulk may spare leukemia stem cells (LSCs) that provide a reservoir of premalignant or malignant clones that can regenerate the tumor. This is of great significance for AML therapy. Most patients who go into remission after treatment will relapse within the first few years, which diminishes their rate of survival substantially. Therefore, safer and more effective therapies are urgently required for the majority of AML patients with severely limited effective treatment options. A better understanding of the molecular landscape of AML and the biology of LSCs may, therefore, aid the design of much more targeted therapies for AML. We will discuss advances in our understanding of these processes in more detail in the following section with a focus the contribution of epigenetic regulators to AML heterogeneity and for the emergence and sustenance of LSCs.

### Epigenetic Regulators and the AML Mutational Landscape

**Figure 1:**
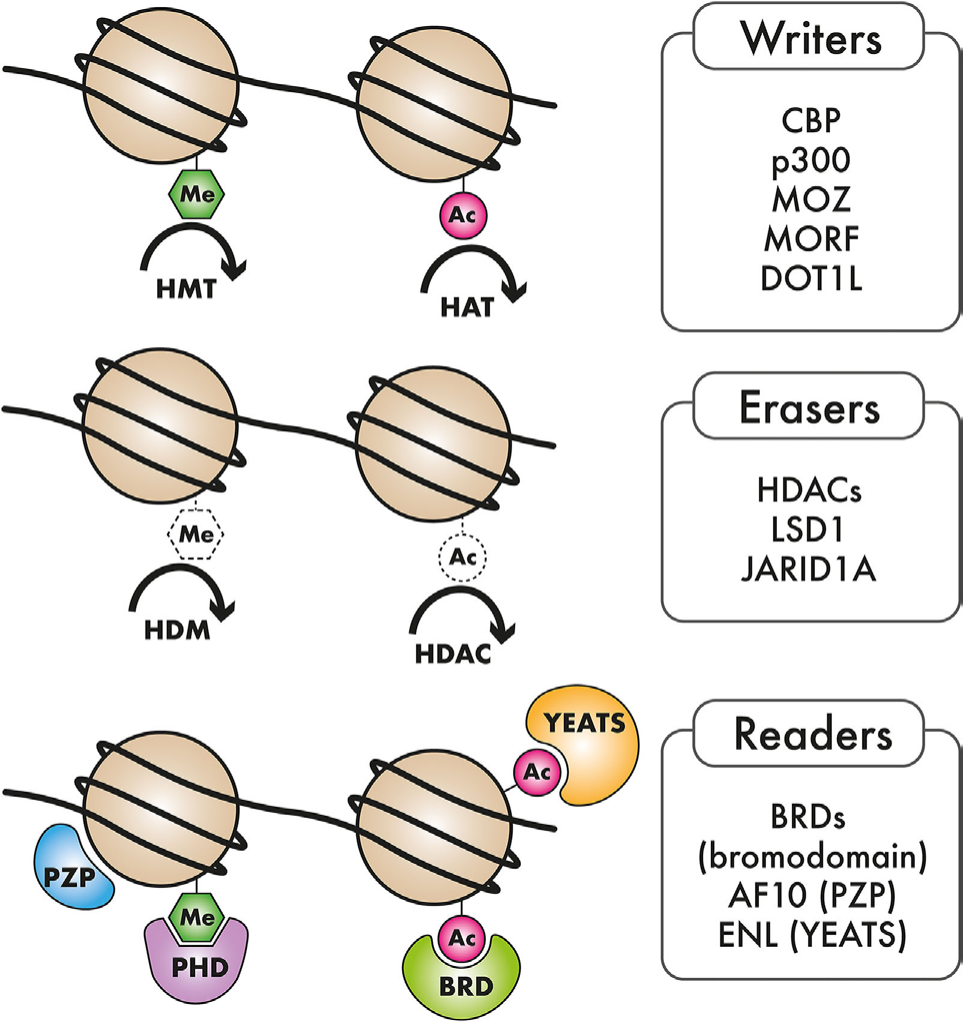
Types of epigenetic regulators mutated in AML: Epigenetic “writers,” such as DNA methyltransferases, histone methyltransferase (HMT), and histone acetyltransferases (HAT), deposit methylation and/or acetylation on DNA or on histones. These epigenetic marks may be removed by epigenetic “erasers,” including histone demethylase (HDM) and histone deacetylase complexes (HDACs). Epigenetic “readers” are highly specialized proteins that specifically bind to distinct epigenetic marks to convey this information to downstream effectors.

Acute myeloid leukemia is highly heterogeneous in terms of its underlying genetics, pathobiology, and clinical manifestation. Even though the morphological and cytogenetic heterogeneity of AML has been recognized for several years, the marked molecular heterogeneity has only come to the fore recently. Emerging evidence from genome-scale studies propelled by advances in next-generation sequencing (NGS) has substantially broadened our knowledge of the spectrum and frequency of mutations in AML. Characterization of the genomic AML landscape has led to the identification of recurrent mutations in a number of previously uncharacterized genes in AML. The classes of genes mutated in AML include transcription factors, kinases, cell cycle regulators, spliceosomal genes, and epigenetic regulators. The observation that genes encoding epigenetic regulators are among the most commonly occurring mutated factors in AML, strongly points to a role of epigenome dysregulation in AML pathogenesis. These mutations in epigenetic regulators encompass a broad spectrum of epigenetic writer, eraser, and reader proteins which will be the focus of this review. The epigenome is dynamically regulated through chemical modification of DNA and RNA as well as the histone proteins around which DNA is packaged. Our genomes harbor a number of enzymes that deposit these chemical marks (writers), or remove them (erasers), dedicated to specific modifications of DNA or chromatin. Proteins with specialized domains that can selectively bind to specific DNA, RNA, or histone modifications (readers) also abound, indicating a well-orchestrated mechanism for relaying epigenetic marks to downstream effectors. The coordinated action of epigenetic reader, writer, and eraser proteins is important for regulation of various cellular processes, including transcription, DNA replication, cell cycle control, and the DNA damage response. Recurrent genomic alterations in epigenetic writer, reader, and eraser proteins, such as DNA methyltransferase 3A (DNMT3A), TET1/2, IDH1/IDH2, EZH2, mixed-lineage leukemia (MLL), NSD1/3, AF10, ENL, and other epigenetic regulators have been cataloged in AML, inspiring a wave of preclinical studies aimed at uncovering causal links between epigenome dysregulation and leukemogenesis (see Figure 1 and Table 1). These studies are yielding important actionable information that can be rationally applied to the development of epigenome-based therapies for AML patients.

**Table 1:** Frequency and role of recurrently mutated epigenetic regulators in acute myeloid leukemia (AML).

Genes (reference)	Frequency in AML	Mutation type	Description
*DNMT3A* (4–6)	~12–22%	Point mutation/indel (~60% R882H)Loss-of-function	DNMT3A mutations cause genome-wide DNA hypomethylation *in vitro* and may have dominant negative effects
*IDH1/IDH2* (7–10)	~10–20%	Missense point mutation (R132-IDH1, R140/172-IDH2)Gain-of-function	Mutants of cytoplasmic (IDH1) and mitochondrial (IDH2) decarboxylase convert isocitrate to 2-HG, which inhibits TET2, result in genome-wide DNA hypermethylation
*TET2* (11–13)	~14%	Point mutation/indelLoss-of-function	A 5-mC-dioxygenase that converts 5-mC to 5-hmC, an intermediary process for demethylation.*TET2* mutations phenocopy *IDH* mutations
*EZH2* (14–16)	<1%	Point mutation/indelLoss-of-function	An enzymatic component of PRC2 and H3K27 methyltransferase. Biological mechanism unclear
MLL-fusion proteins (17–21)	~3–5% PTD/~5–10%	Partial tandem duplication (PTD)/translocationGain-of-function	Duplication of an internal N-terminal region of MLL, retains SET domain/fusions of MLL N-terminal region to several different partner proteins, create dominant transcriptional activators
CBP/p300-MOZ/MORF fusion (22–24)	<1%	TranslocationGain-of-function	Acetyltransferases involved in rare but recurrent chromosomal translocations with elevated *HOX* gene expression and adverse prognosis

*DNMT3A, DNA methyltransferase 3A; Indel, insertion and/or deletion; IDH, isocitrate dehydrogenase; 2-HG, 2 hydroxyglutarate; TET2, tet methylcytosine dioxygenase; 5-mC, 5-methylcytosine; 5-hmC, 5-hydroxymethylcytosine; EZH2, enhancer of zeste homolog 2; PRC2, polycomb repressive complex 2; H3K27, lysine 27 of histone H3; MLL, mixed-lineage leukemia; CBP, CREB-binding protein; MOZ, monocytic leukemia zinc-finger protein; MORF, MOZ homolog; HOX, homeobox*.

### LSCs and the Epigenome

The failure of “debulking” strategies in AML can now at least partly be attributed to AML–LSCs. Several lines of evidence demonstrate that AML emerges from a subset of cells with stem-cell-like properties [reviewed in Ref. (25, 26)]. It is now well documented that long-lived normal hematopoietic stem cells (HSCs) can accumulate mutations bearing the potential to trigger myeloid transformation in later life (27–30). These mutant HSC clones can eventually transform into LSCs, a population of cells with stem-cell properties that have the ability to sustain and propagate the tumor. Alternatively, certain AML-specific mutations in downstream hematopoietic progenitors can also initiate a transcriptional program reminiscent of HSCs, converting them to self-renewing LSCs (31, 32). It is now clear that stemness attributes in cancer are much more fluid than previously imagined, especially in constantly evolving neoplastic cells that display an enormous amount of genetic and epigenetic instability. Therefore, instead of the presence of a fixed, immutable population of cancer stem cells, there is evidence suggesting that cancer cells can switch between stem-like and non-stem-like states within the tumor, making the cancer stem cell a “moving target.” Such extraordinary plasticity of tumor cells requires rapid adaptations to changing micro-environmental cues as well as to the selective pressures mounted by aggressive therapeutic interventions typically used in cancer patients. This exceptional plasticity is likely to be provided by rapid and reversible epigenetic, rather than genetic changes in cancer cells. This is especially likely since epigenetic changes govern key steps in the transition of stem cells to their differentiated progeny in the process of normal hematopoiesis (33). Consistent with this notion, it is no surprise that almost all of the epigenetic regulators with recurrent AML-associated mutations have important roles in HSC self-renewal, survival, or differentiation. Importantly, studies have shown that mutations in epigenetic modifiers, including *DNMT3A* and *IDH1/IDH2*, occur in early pre-leukemic HSCs (29, 34, 35), while signaling pathway mutations in genes that confer proliferative advantage, such as *NPM1* (nucleophosmin 1), *FLT3-ITD* (internal tandem duplication of the *FLT3* gene), and *KRAS/NRAS*, are acquired later during the development of AML (36). Strikingly, there is increasing evidence that mutations that lead to clonal expansion of HSCs are acquired during normal aging, a process that is termed “clonal hematopoiesis.” Individuals with clonal hematopoiesis have an increased risk of progression to myeloid neoplasia and lower overall survival. Interestingly, a large proportion of the mutations observed in normal elderly individuals with clonal hematopoiesis are in epigenetic regulators (27–30). These striking observations indicate that mutations in epigenetic regulators may establish a leukemia-predisposing epigenetic state in premalignant HSC clones. These HSC clones may then be poised to transform into fully leukemic LSCs upon acquisition of secondary mutations with complementary oncogenic activities. Taken together, therapeutic targeting of the epigenome may turn out to be an attractive strategy for targeting AML–LSCs and may provide lasting curative benefit, especially in combination with traditional “debulking” strategies.

## Epigenetic Regulators in AML Pathogenesis

Ever since chromosomal translocations and fusion oncogenes were discovered in AML several years ago, it was apparent that chromatin modulators such as the “writers” MLL1/KMT2A, CBP/p300, and NSD1/KMT3B might have causative roles in AML pathogenesis. MLL1, CBP, and NSD1/3 are involved in recurrent chromosomal translocations in a fraction of AML patients. These translocations were discovered early because they could be observed using methods, such as karyotyping and fluorescence *in situ* hybridization, that enabled identification of gross genetic aberrations in AML cells. However, these chromatin modifier mutations only accounted for a minor fraction of AML patients. There was little evidence for the direct genomic alteration of epigenetic regulators in the vast majority of AML. This scenario changed dramatically with the recent explosion in NGS, whereby mutations in several novel genes not previously implicated in AML pathogenesis were identified. Recent NGS-based discovery efforts in AML have demonstrated that epigenetic regulators comprise one of the most frequently mutated classes of genes in AML, accentuating the role of the epigenome in AML pathogenesis. Recurrent mutations in DNA methyltransferases (*DNMTs*), isocitrate dehydrogenases (*IDH1/IDH2*), methylcytosine dioxygenases of the ten-eleven-translocated (TET) family, and human homologs of the Drosophila polycomb complex such as Enhancer of Zeste 2 (*EZH2*) and additional sex-combs like genes (*ASXL1/2*) have been discovered in AML and myelodysplastic syndromes and myeloproliferative neoplasms (MDS/MPN), and many of these mutations have been causally linked to myeloid transformation in murine models. The role of these epigenetic modifiers in AML pathobiology and studies exploring these proteins as druggable targets will be described in detail below. Apart from genes mentioned above, there are a number of examples of epigenome modulators that are not directly mutated but nevertheless implicated in AML pathogenesis. Several chromatin modifiers have been discovered as selective dependencies in specific AML subtypes as discussed in the Section “*DNMT* Mutations.”

### *DNMT* Mutations

DNA methylation is an important process in development that involves the addition of a methyl group to the carbon-5 position of cytosine in CpG dinucleotides, leading to the formation of 5-methylcytosine (5-mC). The DNMT family, including *DNMT1, DNMT3A*, and *DNMT3B* encode methyltransferases that catalyze this reaction. DNMT3A and DNMT3B are largely *de novo* DNMTs, whereas DNMT1 predominantly plays a role in the maintenance of DNA methylation (37). CpG clusters are enriched in regions upstream of genes (CpG islands) and increased methylation of CpG islands leads to transcriptional silencing of the downstream gene. Recurrent mutations in *DNMT3A* are observed in 12–22% of AML and always present as heterozygous mutations. *DNMT3A* mutations are associated with poor prognosis and decreased overall survival (4). A majority of these mutations lead to premature truncation of DNMT3A protein through nonsense or frame-shift mutations in the protein-coding region. Approximately 60% of *DNMT3A*-mutated AML patients harbor a missense mutation in the arginine 822 residue that diminishes its methyltransferase activity while reducing its binding affinity to DNA, which has been proposed to have a dominant negative function over the wild-type DNMT3A protein (5). *DNMT3A* mutations have been observed in non-leukemic T-cells from AML patients as well as in normal elderly individuals with no signs of leukemia, suggesting their provenance from an early, premalignant multipotent cell (27, 35). The mechanisms of leukemogenesis by DNMT3A are not entirely clear; however, studies have shown that heterozygous *Dnmt3a* ablation in mice leads to an expansion of the HSC pool (38), myeloid skewing and a predisposition to myeloid malignancies that may require additional genetic alterations. These studies reinforce the notion that the *DNMT3A* mutation, perhaps like mutations in other epigenetic regulators, do not lead to frank leukemic transformation on their own, but rather create a premalignant state that lays the ground for malignancy. Recently, it was also reported that mutant DNMT3A (R882H) interacts with the Polycomb repressive complex 1 (PRC1) to silence genes, suggesting that PRC1 activity could be an attractive target in DNMT3A-mutant tumors (39).

### Isocitrate Dehydrogenase (*IDH*) Mutations

Isocitrate dehydrogenases are key components of the tricarboxylic acid cycle responsible for oxidative decarboxylation of isocitrate to α-ketoglutarate (α-KG). The IDH1 and IDH2 proteins are nicotinamide adenine dinucleotide phosphate (NADP+)-dependent enzymes that mediate a number of important cellular processes including lipid metabolism, glucose sensing, and oxidative phosphorylation (7). *IDH1* and *IDH2* mutations are found at a frequency of 10–20%, and these mutations are more common in the cytogenetically normal sub-group of AML. *IDH1* and *IDH2* mutations are mutually exclusive and result in a gain of neomorphic activity (8). Specifically, gain-of-function *IDH* mutations convert the metabolite α-KG to the structurally similar I-2-hydroxyglutarate (2-HG). 2-HG acts as an “oncometabolite” since its accumulation in leukemic cells interferes with the enzymatic functions of several chromatin modifiers that use α-KG as a cofactor. Mechanistic investigations into the model of action of *IDH* mutations have shown that hematopoietic specific IDH1 (R132H) mutation using a conditional knock-in strategy expands HSC and myeloid progenitor compartments but fail to show signs of overt AML (40). Similar results were demonstrated by Heuser and colleagues using a retroviral bone marrow transplantation model which showed that mutant *IDH* overexpression was not sufficient to cause AML, but could do so in the presence of the Hoxa9 oncogene (9). These results suggest that similar to DNMT3A, *IDH* mutations may also need secondary mutations for initiation of frank malignancy in AML. Strikingly, the same group also demonstrated that *in vivo* injection of the oncometabolite 2-HG, could recapitulate most, but not all of the oncogenic effects of the *IDH1* mutation (10). These interesting observations reinforced the role of 2-HG as an oncometabolite but also suggested that IDH1 may have additional oncogenic functions beyond its role in 2-HG accumulation. The exact role of chromatin modifying enzymes and epigenomic modifications in relaying the consequences of *IDH* mutation to oncogenic transcription remains to be determined.

### *TET* Family Mutations

One of the most important classes of enzymes affected by *IDH* mutations is the TET family of methylcytosine dioxygenases. Normally, TET2, with the cofactor molecule α-ketoglutarate (α-KG), converts 5-mC to 5-hydroxymethylcytosine (5-hmC), which can then be demethylated back to cytosine *via* a series of intermediate steps (11) This TET-enzyme catalyzed CpG demethylation is an important step in the dynamic regulation of DNA methylation associated regulation of cellular processes. Inactivating mutations in TET enzymes lead to decreased hydroxylation of methyl-CpG sites (12, 41) resulting in aberrant CpG hypermethylation, decreased expression of key differentiating enzymes, and inhibition of normal cellular differentiation (42). Several studies have examined the function of TET2 inactivation in mice, *Tet2* deletion leads to hematopoietic defects including enhanced HSC self-renewal and myeloid expansion, correlating with global loss of 5-hmC in primitive hematopoietic populations (43–45). It was recently described that restoration of TET function using an inducible shRNA model of TET-induced AML or through the administration of Vitamin C, which is a cofactor for α-KG dependent dioxygenases reverses leukemogenicity induced by the mutant TET protein (13). These exciting results imply that metabolic control of TET activity could be harnessed for therapeutic benefit in patients with TET mutations. Notably, cytosine methylation signatures of *TET2*-mutated AML show significant overlaps with those found in *IDH1/IDH2* mutated patients and *IDH1/IDH2* and *TET2* mutations are mutually exclusive in AML (8), signaling a common mechanism of leukemogenesis based on aberrant DNA methylation. Recently another addition to this sub-group was made due to the discovery that mutations in the Wilms tumor gene *WT1*, which are found in approximately 10% of AML, are also mutually exclusive with *TET* and *IDH* mutations and display global cytosine hydroxymethylation profiles reminiscent of *IDH* and *TET* mutated AML. Levine and colleagues, who reported these observations, went on to demonstrate that WT1 physically interacts with TET proteins, TET2 and TET3, and compromises TET functions. It was concluded in this study that *IDH1/IDH2, TET2*, and *WT1* mutations define a common AML subtype with overlapping disordered DNA 5-hmC profiles (46). Taken together, these results imply that dysregulated DNA methylation, achieved either through mutations in *DNMT3A, IDH1/IDH2, TET2*, or *WT1*, play an important role in the pathogenesis of a large proportion of AML patients. This information may help identify common targeted therapies for patients with mutations in these functionally related genes.

### *MLL/KMT2A* Tandem Duplications

The *MLL/KMT2A* gene was one of the first epigenetic regulators known to be involved in leukemia pathogenesis. MLL is a chromatin writer, a SET-domain containing lysine methyltransferase belonging to the *Drosophila* Trithorax family of proteins. Approximately 3–5% of *de novo* AML present with in-frame partial tandem duplications of MLL exons 3–9 or 3–11 (17). This mutation is associated with a poor prognosis (18, 19). The MLL partial tandem duplication (MLL-PTD) duplicated the N-terminal AT-hook region of MLL, in addition to a domain that preferentially binds to unmethylated CpG sites and a transcriptional repression domain (20, 21). Mice carrying the *MLL-PTD* mutation show developmental abnormalities and dysregulated *Hox* gene expression similar to AML patients with the *MLL-PTD* mutation (47), but require additional leukemogenic driver mutations such as the Flt3-internal tandem duplication (48) for overt leukemogenesis. Intriguingly, a recent study from Koeffler and colleagues aimed at capturing the mutational landscape of *MLL-PTD* AML demonstrated that *MLL-PTD* mutations co-occur with several other mutations, including *FLT3-ITD, DNMT3A, IDH1, TET2*, cohesion genes, and splicing factors, but not *NPM1* which is the most commonly mutated gene in AML (49). These studies suggest that *MLL-PTD* and *NPM1* mutations may act through overlapping mechanisms. Furthermore, ordering of mutations in this study suggested that the *MLL-PTD* mutation was a secondary mutation that was undetected in remission in contrast to persistent mutations in epigenetic regulators, such as IDH2/DNMT3A and TET2.

### *MLL/KMT2A* Translocations

In addition to tandem duplications of MLL that are observed in AML, the chromosomal band 11q23 is also involved in chromosomal translocations that fuse MLL to a partner gene on another chromosome. MLL fuses to several different partner genes; more than 80 different MLL-fusion partners have been discovered to date (50, 51). MLL-fusions are observed in 5–10% of adult AML and approximately 15–20% of AML in infants (50). In infant ALL, the frequency of MLL-rearrangements is as high as 70% (50), highlighting the role of these fusions in leukemogenesis. The binding of MLL-fusions to their target promoters is contingent upon the interaction of the N-terminal part of MLL with the LEDGF protein, an interaction that is bridged by the protein Menin (MEN1). The MLL–Menin interaction, therefore, is an attractive target for therapy and small-molecule compounds targeting this interaction have been developed (52–55). MLL-fusion protein expression activates a cascade of downstream transcriptional programs, one of the most important of which is the clustered homeobox (*HOX*) genes and their cofactor MEIS1. These *HOX/MEIS* genes are crucial for perpetuating the highly self-renewing state that is triggered by MLL-fusion protein expression in transformed hematopoietic progenitors. Indeed, several recent studies have shown that oncogenesis by MLL-fusion proteins requires the coordinate action of a number of chromatin factors that are essential and rate limiting for the transcriptional activation of *HOX/MEIS* genes. A prime example of this is the histone methyltransferase (HMT) DOT1L. The DOT1L protein biochemically interacts with several of the most common MLL-fusion partners, including AF4, AF9, ENL, AF10, and AF17 (56–59). All of these fusion partners retain the DOT1L interacting motif in their respective MLL-fusion events, and this interaction has been shown to be necessary and sufficient for oncogenic transcriptional activation functions by MLL-fusion proteins. Based on structure–function assays, genetic studies, and small-molecule inhibitor investigations, DOT1L has emerged as a clear therapeutic target in MLL-rearranged AML and clinical trials are currently ongoing (60) as described later in the review. Interestingly, DOT1L seems to be generally involved in *HOX/MEIS* regulation and other models of AML where *HOX/MEIS* activation is observed are sensitive to genetic and/or pharmacological DOT1L inhibition. These include AML driven by MLL-fusion proteins that do not recruit DOT1L, MLL-tandem duplications, nucleoporin 98 (NUP98)–NSD1 fusions, *NPM1* mutations or mutations in the *DNMT3A* gene (59, 61–63). Strikingly, MLL–Menin inhibitors also seem to show broad activity against diverse HOX-activating AML oncogenes, suggesting that both these proteins are involved in an epigenetic network that is broadly essential for sustaining HOX gene expression (61). MLL-fusion transformed cells have also been shown to be sensitive to the depletion of several other chromatin factors, including PRC1 and polycomb repressive complex 2 (PRC2) complex proteins (64–69), the histone acetyltransferases (HATs) MOF, the arginine methyltransferase PRMT1 (70), and the MLL methyltransferase paralog MLL2 (71). Another interesting aspect of MLL-rearrangements is the involvement of chromatin readers. Many of the common fusion partners of MLL have chromatin-reading domains that recognize specific histone modifications and these reader–histone interactions and their transcriptional consequences are only recently being uncovered. AF9 and its paralog ENL harbor YEATS domains in their N-terminal region that bind to specific acetylated or crotonylated histone residues (72, 73). AF10 and AF17 on the other hand, have N-terminal PHD-zinc finger-PHD (PZP) domains that specifically recognize unmethylated H3K27 (74). Even though the chromatin reader modules of these MLL-fusion partners are excluded from MLL-fusion proteins themselves, chromatin reading by some of the wild-type, non-rearranged MLL-fusion partners, such as AF10 and ENL, have been shown to be important for MLL-leukemogenesis (73–75). Intriguingly, MLL-rearranged AML cells, which were dependent on AF10 or ENL for their proliferation, were found to be insensitive to the inactivation of their closely related paralogs AF17 or AF9, respectively. Even though this mystery of differential sensitivity is still unresolved, the fact that chromatin reading by specific PZP and YEATS domains are critical for MLL-leukemogenesis opens up the exciting possibility of targeting MLL-rearranged leukemias using selective small-molecule inhibitors of these chromatin-reading modules that are likely to be developed in the near future.

### PRC Dysregulation in AML

Polycomb group (PcG) proteins are transcriptional repressors that regulate key fundamental processes, including cellular identity, differentiation, and stem cell plasticity (76). PcG proteins have highly conserved roles throughout evolution in the silencing of transcription through specific histone modifications. PcG proteins are constituents of two major multi-subunit complexes, PRC1 and 2, which have distinct effects on chromatin, gene expression, and developmental regulation. The PRC2 complex consists of four core constituents: The *Drosophila* enhancer of zeste homolog (EZH2), embryonic ectoderm development, suppressor of zeste homolog, and RbAp46/48, also known as RBBP4. PRC1 composition is more variable with only two core components RING1A and RING1B which complex together with the proteins BMI1, MEL18, or NSPC1 (76). The PRC2 complex is involved in histone 3 lysine 27 mono, di, and trimethylation, a function that shows high evolutionary conservation as a major facilitator of gene silencing. EZH2, the enzymatic component of PRC2 is mutated in myeloid malignancies, most commonly in MDS, chronic myelomonocytic leukemia (CMML), and primary myelofibrosis and rarely in AML (14–16). These mutations are missense or frame-shift mutations, which are predicted to lead to EZH2 loss of function. Interestingly, in diffuse large B-cell lymphoma (DLBCL), approximately 20% of patients bear activating *EZH2* mutations (77), suggesting that PRC2 may have contrasting context-dependent roles in oncogenesis. Wild-type *Ezh2* depletion in murine hematopoietic progenitors leads to myeloproliferative effects (78), whereas depletion of non-enzymatic PRC2 components such as *Eed* leads to severe lethal myelo- and lympho-proliferative disorders (79). These results indicate that further investigations are required to clarify the roles of EZH2 and PRC2 activity in leukemogenesis.

Of the PRC1 components, the *BMI1* oncogene is implicated in the self-renewal of normal as well as leukemic stem cells in AML (80). Despite the apparent importance of BMI1 in normal and leukemic stem cells, mutations in this PRC1 component or any other members of the PRC1 complex have not been identified in AML.

### Demethylase Mutations

Mutations in the histone 3 lysine 27 demethylase *UTX* are found in a variety of human cancers, including multiple myeloma, esophageal squamous cell carcinomas, and renal cell carcinoma (81). In myeloid malignancies, *UTX* mutations are found in 8% of patients with CMML and approximately 10% of patients with CMML-derived secondary AML. Most of these mutations were adjacent to the Jumonji C domain of UTX, which is required for the demethylase activity of UTX, suggesting that UTX loss of function may contribute to leukemogenesis. The JARID1A (KDM5A) H3K4 demethylase is fused to NUP98 in approximately 10% of pediatric acute megakaryoblastic leukemia resulting in the cytogenetically cryptic NUP98–JARID1A translocation. These fusions are believed to compromise normal functions both of NUP98 as well as JARID1A, leading to leukemogenesis (82). Exact consequences of demethylase mutations in these rare AML subtypes and their role in leukemogenesis remain to be discovered.

### NSD Gene Fusions

Nuclear receptor-binding SET domain protein 1 is a HMT that is involved in recurrent chromosomal translocations with the NUP98 gene that are usually cryptic. NUP98–NSD1 fusions are found at a significantly increased frequency in pediatric as compared to adult patients (approximately 5 vs 1.4% of AML, respectively) (83, 84). In both adult and pediatric AML, NUP98–NSD1 translocations confer a poor prognosis and are enriched in the cytogenetically normal AML cohort. Mechanistically, NUP98–NSD1 fusions drive abnormal expression of *HOX/MEIS* oncogenes and this activation is dependent on the H3K36 methyltransferase activity of NSD1. NSD1-driven H3K36 methylation repels PRC2 complex proteins from the HOX/MEIS and other NUP98–NSD1 target genes, leading to sustained transcriptional activation and oncogenesis. NUP98-fusions with NSD3, a close homolog of NSD1 have also been reported in AML (85), further highlighting the role of this family of proteins in AML pathogenesis.

### CBP/p300 and MOZ–MORF Fusions

The *mo*nocytic leukemia *z*inc-finger MOZ (MYST3) protein and its paralog MORF (MYST4) are HATs involved in recurring chromosomal rearrangements in AML. The balanced chromosomal translocation t(8;16)(p11;p13), which is found in <1% of AML patients, leads to in-frame fusions of MOZ with the HAT CBP (22). Another common partner of MOZ is TIF2, a member of the p160 family of nuclear receptor co-activators (86). MOZ-TIF2 expression in murine hematopoietic progenitor cells leads to aberrant *Hoxa* gene activation, increased self-renewal, and transformation in *in vitro* and *in vivo* assays. Notably, TIF2 interacts with CBP, indicating a common thread that links MOZ-fusions is the enlisting of CBP/p300 HAT activity. Consistent with this notion, MORF–CBP fusions, as well as fusions of either MOZ or MORF to the CBP homolog p300 have also been observed in AML, signifying common mechanisms linking these paralogous pairs of HATs to leukemogenesis. *HOX* gene activation is also observed in AML cells bearing MOZ–CBP fusions, similar to MLL and NUP98-fusion proteins (23). Even though patterns of *HOX* gene activation vary depending on which *HOX*-activating fusion protein is present in AML cells, *HOX* gene activation seems to be causally linked to transformation in all these AML subtypes based on preclinical studies.

### Hijacking of Chromatin Modulators by AML Oncogenes

Apart from the epigenetic regulator mutations mentioned above, there are a number of examples of epigenome modulators that are not directly mutated but nevertheless implicated in AML pathogenesis. In recent years, several chromatin modifiers have been discovered as selective dependencies in specific AML subtypes as discussed briefly in the Section “The Advantage of Epigenetic Therapies.” Some of the most striking examples of epigenetic regulator hijacking for AML pathogenesis are observed in studies with oncogenic fusion proteins. Co-option of histone methyl and acetyltransferases, such as DOT1L by MLL-fusion proteins has been discussed in detail in the Section “CBP/p300 and MOZ–MORF Fusions.” In addition, a number of AML fusion proteins interfere with functioning of the PRC1 and PRC2 complexes. The promyelocytic leukemia–retinoic acid receptor (PML–RAR) fusion, which is seen in approximately 95% of the cases of acute promyelocytic leukemia (APL) (87, 88) can participate in biochemical interactions with several PRC2 complex proteins, recruiting repressive epigenetic modifications on target loci, while the other PML fusion oncoprotein PLZF–RARA binds to PRC1 complex members (89, 90). In separate studies, the PML–RARA fusion protein has also been shown to enlist the gene silencing activity of DNMT3A and HDAC3 complexes through biochemical interactions with the fusion protein (91–93). Similarly, the AML1/ETO fusion protein, a product of the recurrent t(8;21)(q22;q22) translocation, one of the most common cytogenetic abnormalities in AML, participates in biochemical interactions with chromatin modulatory proteins. AML1–ETO interacts with the protein arginine methyltransferase PRMT1. PRMT1 knockdown reduces the transcription of AML1–ETO target genes, implicating PRMT1 activity in AML1–ETO pathogenesis (94). AML1–ETO also acts as a transcriptional repressor and the repressive mechanisms of AML1–ETO have been shown to be facilitated by biochemical interactions with repressive complexes, such as N-CoR, mSin3A, SMRT, and HDAC1 (95–101).

## The Advantage of Epigenetic Therapies

The last few years have seen a wave of unprecedented activity in the development of novel therapeutic agents and treatment strategies for AML. These include novel monoclonal antibody-based therapies, potent small-molecule inhibitors of signaling pathway mutations, tyrosine kinases, nuclear export, and immunotherapy. Most of these approaches are guided by specific mutations found recurrently in AML patients, which may herald a new era of precision medicine in AML. This strategy has been used with great success for more than a decade in the treatment of chronic myeloid leukemia and APL, but has largely failed in AML due to the absence of a single defining mutation event or hitherto intractable molecular targets. The recurrent prevalence of epigenetic regulator mutations in subsets of AML as well as broad epigenomic reprogramming across AML subtypes has ignited vigorous efforts to therapeutically target the AML epigenome. One of the biggest advantages of exploiting the epigenome as a therapeutic target is that, in contrast to the genomic alterations observed in AML cells that are difficult to reverse, epigenetic abnormalities can be reverted using pharmacological agents. Many epigenetic regulators such as DNA and histone modifying proteins have enzymatic activity, which is considered more amenable to therapeutic targeting using small-molecule inhibitors than other classes of proteins such as transcription factors. Another consideration is that since mutations in chromatin modulators are often observed in founding AML clones, targeting mutated epigenetic regulators may also eliminate LSCs, thereby striking at the root of AML and prevent relapse. For all of these reasons, the AML epigenome has emerged as one of the most exciting frontiers for drug discovery in recent years. Recent advances in preclinical and clinical development of epigenome-based therapies in AML will be discussed in the Section “Emerging Epigenome-Based Therapies in AML.”

### Emerging Epigenome-Based Therapies in AML

**Figure 2:**
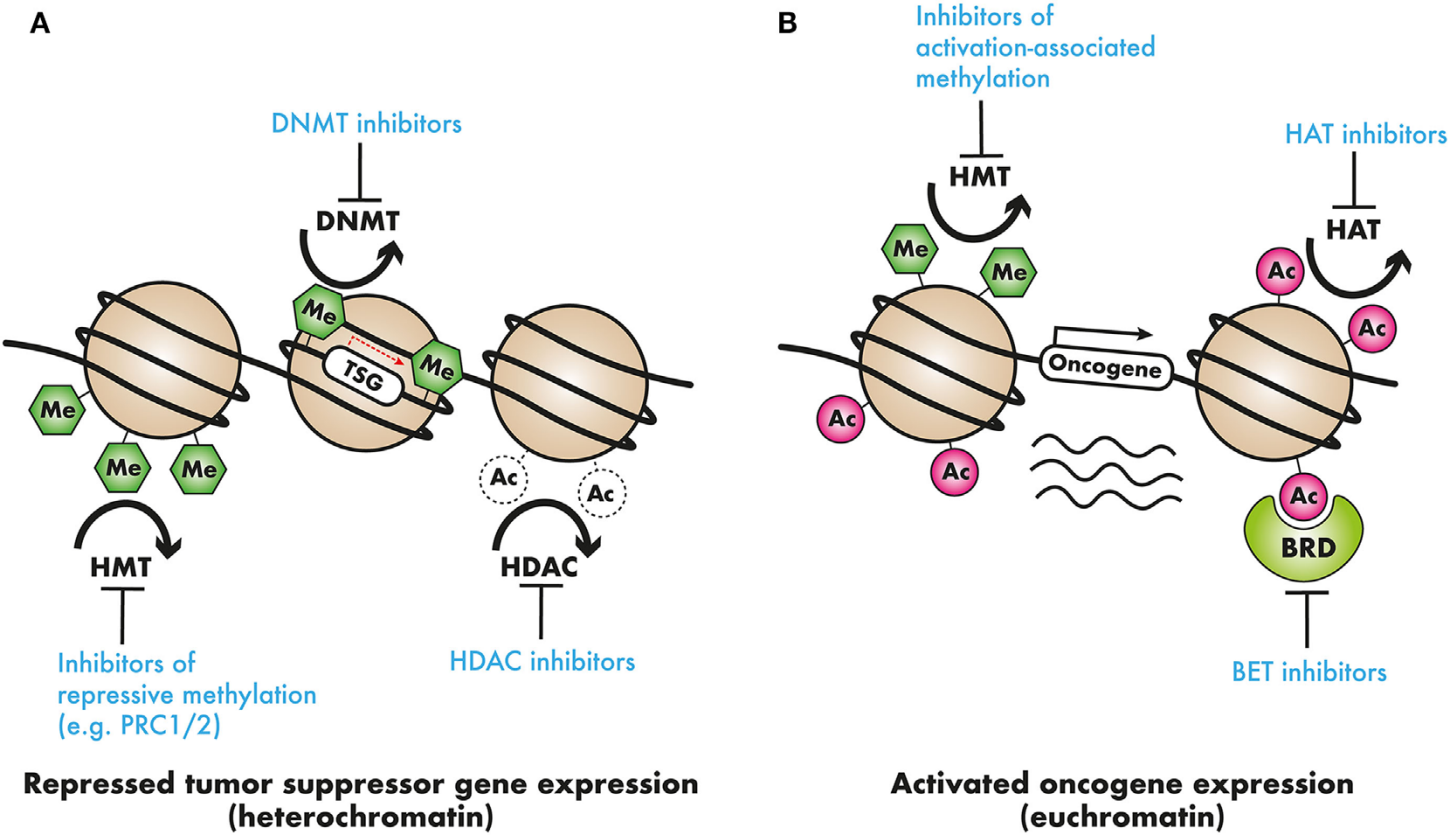
Epigenetic modifiers in cancer as clinical targets: **(A)** tumor suppressor genes (TSG) may be silenced by chromatin compaction resulting from DNA methylation or histone deacetylation, or repressive histone methylation. DNA methyltransferase (DNMT) inhibitors, demethylating agents, histone deacetylase (HDAC) inhibitors, or inhibitors of repressive histone modifying complexes such as PRC2 may restore the expression of these TSGs. **(B)** In contrast, oncogene activation by means of activation-associated histone hypermethylation, or histone hyperacetylation could be countered with the use of selective histone methyltransferase (HMT) or acetyltransferase histone acetyltransferase (HAT) inhibitors. Additionally, readers that recruit these activation-associated marks, such as the AF10 PZP domain, the AF9 or ENL YEATS domain, and the BRD4 bromodomains, and recruit transcriptional complexes present targets for pharmacological intervention.

Some of the early epigenome-based strategies have focused on broad-based epigenomic reprogramming aimed at restoring the altered epigenomic configurations in AML cells. This kind of broad epigenomic reprogrammig—for example, with the use of DNMT or histone deacetylase complex (HDAC) inhibitors—has been shown to reverse the commonly observed silencing of tumor suppressor genes (TSG) and restore normal differentiation. Since these epigenetic processes are involved in both silencing as well as activation of transcription dependent on the epigenetic mark and the chromatin context, it may be very difficult to identify which subset of AML may benefit most from broad-based epigenomic reprogramming therapies. More targeted therapies require the identification of specific silenced TSG or activated oncogenes for targeted therapeutics (Figure 2). Nevertheless, broad-based inhibition of DNA methylation and histone deacetylation using DNMT and HDAC inhibitors has been explored extensively as a therapeutic strategy in AML. The DNMT inhibitors azacitidine (AZA) and decitabine (DAC) are extensively used in MDS and also in patients with AML, where they show benefit, especially in elderly AML patients (102). Drugs, such as valproic acid (VPA), panobinostat and vorinostat, are some of the HDAC inhibitors approved for clinical use. More recently, after the identification of epigenetic regulator mutations, efforts have intensified to precisely target the oncogenic activity of those mutant proteins. This approach is particularly promising, as it may finally lead to precisely targeted therapies in patients with non-APL AML. Finally, as mentioned previously, there is compelling evidence that some AML-activated oncogenic transcriptional programs are specifically dependent on chromatin regulatory proteins, marking these chromatin regulators as attractive candidates for therapy. Prominent examples are the HMT DOT1L that regulates *HOX* gene expression and the bromodomain-containing protein BRD4, which regulates the expression of super-enhancer linked genes in AML and other cancers. These newly discovered dependencies present hitherto unexplored epigenetic vulnerabilities for therapeutic intervention (see Table 2). In the Section “Broad Epigenomic Reprogramming As a Therapeutic Strategy in AML,” pharmacological strategies that employ broad epigenetic reprogramming, specific targeting of mutated epigenetic regulators, or selective inhibition of cancer-specific epigenetic vulnerabilities will be discussed.

**Table 2:** Current status of select pharmacological agents targeting epigenetic regulators.

Epigenetic target	Agent (reference)	Clinical trials	Mechanism
DNMT3A	Azacitidine (103, 104) Decitabine (105) Guadecitabine (106)	Phase 3 Phase 2/3 Phase 3	Nucleoside analogs that incorporate into DNA to inhibit DNMTs and prevent hypermethylation of tumor suppressor genes (TSG)
HDAC	Panobinostat (107) Vorinostat (108) Entinostat (109) Mocetinostat (110)	FDA FDA Phase 3 Phase 2	Reduction of oncogene transcription and signaling to promote cell cycle arrest and apoptosis
BET	OTX015 (111) INCB054329 (112) FT-1101 (113) GSK525762 (114)	Phase 1/2 Phase 1/2 Phase 1 Phase 1	Reversibly bind to BRDs of BET proteins to prevent acetylated histone binding and inhibit enhancer-mediated oncogene expression
IDH1/IDH2	AG-120 (115, 116) Enasidenib (117, 118)	Phase 3 FDA	Inhibition of mutant IDHs to restore TET2 activity and reduce DNA hypermethylation
EZH2	CPI-1205 (119) Tazemetostat (120)	Phase 1 Phase 1/2	Inhibition of H3K27 methylation to induce apoptosis or differentiation
DOT1L	EPZ-5676 (121, 122)	Phase 1	Inhibition of H3K79 methylation and induces synthetic lethality to cells with MLL rearrangement
LSD1	GSK2879552 (123, 124)	Phase 1/2	Inhibition of H3K4 and H3K9 demethylation to facilitate TSG expression and cell differentiation
MLL–Menin	KO-539 (125)	Preclinical	Selective inhibition of MLL-rearranged cell growth

*DNMT3A, DNA methyltransferase 3A; HDAC, histone deacetylases; BET, bromodomain and extra-terminal motif; BRD, bromodomain; IDH, isocitrate dehydrogenase; TET2, tet methylcytosine dioxygenase; EZH2, enhancer of zeste homolog 2; H3K27, lysine 27 of histone H3; DOT1L, disruptor of telomere silencing 1-like; H3K79, lysine 79 of histone H3; MLL, mixed-lineage leukemia; LSD1, lysine-specific demethylase 1A; H3K4, lysine 4 of histone H3; H3K9, lysine 9 of histone H3*.

#### Broad Epigenomic Reprogramming As a Therapeutic Strategy in AML

##### DNMT Inhibitors

DNA methylation is dysregulated in most cancers including leukemia and has been the preferred target for cancer therapy since the development of hypomethylating agents (HMA). The HMAs AZA and DAC are nucleoside analogs and inhibitors of the DNMT enzymes DMNT1 and DMNT3. Investigational treatment with AZA and DAC in AML started more than 40 years ago [reviewed in Ref (102).]. AZA and DAC are now established as standard options for the treatment of older patients who do not tolerate standard intensive therapy. HMAs are thought to reactivate epigenetically silenced TSG through hypomethylation. Interestingly, HMAs seem to act indirectly through epigenetic reprogramming, rather than through direct cytotoxicity, as indicated by the delayed and prolonged responses (126, 127). Yet, a few caveats exist to the first-generation HMAs. Primary and secondary resistance to HMAs has been commonly reported (128, 129) and both AZA and DAC are degraded in plasma by the enzyme cytidine deaminase. This has promoted the development of second-generation HMAs with enhanced pharmacology and pharmacodynamic properties like guadecitabine, which has shown encouraging results in early clinical trials (130). Even though HMAs have provided much-needed options for older patients, their efficacy as single agent is limited. A number of studies have reported successful early findings from combination trials with HMAs with other agents used in AML such as tyrosine kinase inhibitors (102).

##### HDAC Inhibitors

Histone deacetylase complex inhibitors were initially identified in screens aimed at identifying factors that induce differentiation in leukemia cells (131). Histone acetylation is a major epigenetic mechanism that is carefully maintained by the interplay of HDACs and HATs (132). HDACs enzymatically remove the acetyl group from histones to serve as critical regulators of gene expression. Besides histones, many non-histone proteins that can be reversibly acetylated have been identified and are reported to be involved in a wide range of cellular processes, including gene expression, translation, DNA repair, metabolism, and cell structure (133). Many of these acetylated proteins are known to play roles in tumorigenesis, tumor progression, and metastasis (134). Along with HMAs, histone deacetylase inhibitors (HDACi) were the first epigenetically targeted inhibitors to be FDA approved for the treatment of cancer in the United States. HDAC inhibitors were historically identified based on their ability to induce tumor cell differentiation (135). Inhibition of class I HDACs targets expression of genes involved in cell cycle protein expression, cell cycle arrest in the G2/M phase, and apoptosis. HDAC inhibitors may help reactivate epigenetically silenced TSG including p21 and TP53. VPA was investigated in AML as the inhibitor of class I histone deacetylases. Unfortunately, the response rates of VPA for monotherapy in AML have been relatively low. Several other HDACi have been also tested as monotherapy in myeloid cancers, including romidepsin/depsipeptide (136, 137), entinostat (138), and mocetinostat (139). Likewise, these were found to be insufficient to further develop as a single agent in AML with the overall response rate ranging from 0 to 16%, with transient blast clearance and hematological improvement. Instead, when used in combination with agents with known antileukemia activity, including DNMTi (e.g., AZA, DAC) and chemotherapies, HDACi have shown a decreased time to response and an increase of overall response (107, 108, 140–150). Combination therapy based on the second-generation HDACi vorinostat or entinostat yielded an increased complete remission rate as compared to historical controls (151). The second-generation pan-HDACi panobinostat modulates gene expression by inducing hyperacetylation of core histone proteins, H3 and H4, and was shown to exhibit antitumor activity against several hematologic tumors, both *in vitro* and *in vivo* (107, 152). Even though potent and orally bioavailable, panobinostat yields modest result as a single agent in elderly patients with AML. Adding non-selective HDACi to combination schedules often results in increased toxicities which can lead to dose reduction and early treatment discontinuation (144, 153–157). Therefore, isozyme-selective HDACi with improved safety profiles may overcome this hurdle and provide additional clinical benefit to patients.

##### Bromodomain and Extra-Terminal Motif Protein (BET) Inhibitors

The bromodomain and extra-terminal (BET) protein family serve as transcriptional adapter molecules that facilitate transcription (158–160). They comprise bromodomain-containing protein (*BRD*) *2, BRD3*, and *BRD4*, which are universally expressed, while *BRDT* expression is limited to the testes (160, 161). Diverse functions of BET proteins include histone modification to chromatin remodeling and ultimately lead to transcriptional activation (162) and are essential for cellular homeostasis (160, 163–166). The most well-characterized function of BET proteins is their binding to acetylated lysine restudies through tandem N-terminal bromodomains, These bromodomains are specialized epigenetic reader modules that are essential for high-level expression of oncogenes such as Myc by promoting enhancer activity (167, 168). Recently, they have also been implicated in transcriptional dysregulation in many cancer types, with BRD4 identified as a key player in AML (167, 169–172). BET inhibitors (BETi) reversibly bind the bromodomains of BET proteins. In a variety of human AML cell lines, suppression of BRD4 was shown to suppress MYC effectively suggesting a potential target for cancer treatment (111, 172). OTX015, a thienotriazolodiazepine, is a small-molecule oral inhibitor of BRD 2/3/4 demonstrated to induce apoptosis in a variety of leukemia cell lines and human AML samples (173). BETi have raised great interest as a novel treatment approach, and ongoing phase 1 trials are investigating their single-agent activities along with combination therapies with other novel agents.

##### Lysine-Specific Demethylase 1 (LSD1) Inhibitors

Lysine-specific demethylase 1 has emerged as a promising therapeutic target in multiple cancers, notably in AML (174–179). Its main role is demethylation of H3K4me1/2 and H3K9me1/2 and LSD1 has been shown to dynamically affect a wide range of transcriptional programs in a context-specific manner, acting either as a transcriptional repressor or as an activator (180–183). Pharmacologic inhibition or genetic knockdown of LSD1 in human leukemia cells induces differentiation (123). GSK2879552, an oral LSD1 inhibitor, is currently being investigated as a monotherapy in a phase 1 study for patients with relapsed/refractory AML (NCT02177812). In leukemia cell lines, there appears to be synergism between HDAC and LSD1 inhibitors which supports a clinical trial for further exploration (124). To date, the only HDACi to be evaluated preclinically in combination with an LSD1 inhibitor (SP2509) in AML is the pan-HDACi panobinostat. Treatment with SP2509 and panobinostat resulted in synergistic *in vitro* cytotoxic effects and significantly improved the survival of mice engrafted with AML cells without overt toxicity (178).

##### EZH2 Inhibitors

As mentioned previously, the exact role of EZH2 in AML is not entirely clear. Studies using an MLL-AF9 leukemia model have shown that PRC2 activity is required for MLL-rearranged AML. Inactivation of *Eed*, the critical component of PRC2 prolonged survival and reduced tumor burden in leukemic mice (22). These results were recapitulated with the use of UNC1999, a small-molecule inhibitor of both EZH1 and 2 which upregulated PRC2 target genes such as p16 and p19 in MLL-rearranged leukemia cells and strongly suppressed transformation (64, 184). A number of potent and selective EZH2 and PRC2 inhibitors are being tested in clinical trials in other malignancies where PRC2 activity has demonstrated proto-oncogenic roles such as DLBCL and synovial sarcoma, and it remains to be studied which subsets of AML may benefit from PRC2 antagonist therapies.

#### Targeting of Mutated Epigenetic Regulators

##### IDH Inhibitors

Given the high prevalence of *IDH* mutations in AML as well as in low-grade glioma, intensive efforts are on to develop clinical-grade IDH inhibitors. AGI-6780, a potent and selective allosteric inhibitor of the IDH2-R140Q mutations was recently reported to significantly induce differentiation in primary AML cells bearing IDH2-R140Q in *ex vivo* cultures. More recently another potent small-molecule inhibitor AG-221 (enasidenib) was developed that was shown to confer significant survival benefits in a mouse model of IDH mutant leukemia and also in a xenografts model of primary human AML (185). These exciting studies catapulted IDH inhibitors into clinical trials with very encouraging results, leading to the FDA approval of AG-221 for the treatment of patients with relapsed or refractory AML with *IDH* mutations. Considering that the IDH mutations were only first discovered less than 10 years ago (186), the fact that IDH inhibitors have already been approved for use is an astonishing success story for precision medicine in AML, although the long-term benefits of IDH inhibitors for AML patients remain to be seen.

#### Targeting Epigenetic Dependencies

##### DOT1L Inhibitors

An S-adenosyl-methionine competitive inhibitor of DOT1L (EPZ-4777) was developed by Epizyme Inc. as a potent and selective inhibitor of the methyltransferase activity of DOT1L (187). Using this compound as a tool, several studies preclinical studies were performed to show that MLL-rearranged AML was highly sensitive to pharmacological DOT1L inhibition (59, 187–190). Subsequently, using structure-guided design and optimization of a series of aminonucleoside compounds, the small-molecule EPZ-5676 was developed as a more potent DOT1L with better pharmacological properties than EPZ-4777 (92). Preliminary studies demonstrated potent single-agent antitumor effects of EPZ-5676 in preclinical models of *MLL*-rearranged AML, and synergistic effects with other standard chemotherapeutic drugs (63, 191). EPZ-5676 is being evaluated in clinical trials for adult and pediatric patients with relapsed or refractory AML with *MLL*-rearrangements (122, 187). EPZ-5676 was well-tolerated in initial studies and showed efficacy in a few patients, but several other patients showed moderate to no response, possibly due to pharmacokinetic limitations of the drug. Continued investigation of EPZ-5676 in patients with *MLL* gene rearrangements is warranted and results from the Phase I/II trials are awaited. Next-generation DOT1L inhibitors with improved pharmacological properties are being developed and are likely to show more pronounced efficacy in the clinic.

##### MLL–Menin Inhibitors

The MLL–Menin interaction is retained in all MLL-fusion proteins (192–195). Preclinical studies have demonstrated a critical role for Menin in leukemic transformations mediated by numerous MLL-fusion proteins. Genetic disruption of the MLL–Menin fusion protein interaction abrogates oncogenic properties of MLL-fusion proteins and blocks the development of acute leukemia *in vivo* (195). Recently, small-molecule inhibitors of the MLL–Menin interaction MI-463 and MI-503 were developed, and they were used to demonstrate that pharmacologic inhibition of the MLL–Menin interaction blocks progression of MLL leukemia *in vivo* without impairing normal hematopoiesis (54). These studies have prompted the development of more potent clinical-grade MLL–Menin inhibitors.

## Concluding Remarks

These are still early days for targeted epigenetic therapies, but the prospects are very exciting. There are several challenges ahead that warrant consideration before epigenetic therapies become the mainstay of AML treatment strategies. First, a lot more needs to be done in terms of preclinical and basic research in order to define exact consequences of epigenetic regulator mutations that have been discovered in AML. This will require the development of faithful genetically engineered mouse models that recapitulate AML mutations, combined with detailed studies on normal and leukemic hematopoiesis. Characterization of the impact of these mutations on normal physiological processes in general and hematopoiesis, in particular will be helpful in predicting potential toxicities. Second, barring few exceptions, it is not entirely clear which subsets of AML may benefit from a particular epigenome-based therapy. Matching patients appropriately to epigenetic therapies will require detailed characterization and sensitivity studies including *in vitro* and *in vivo* inhibitor or genetic screens or epigenomic studies aimed at identifying specific “epigenetic lesions” and their respective drivers. Finally, there is an urgent need for the development of more potent and more selective small-molecules targeting epigenetic regulators. This is a rapidly developing field and selectively small-molecule inhibitors of class-specific HDACs, HATs, as well as DNA and HMT are being developed by several academic investigators and pharmaceutical companies. The next decade will see unprecedented activity in preclinical and clinical investigation of epigenome-based therapies.

## Author Contributions

AD, YS, and BC conceived and drafted the manuscript. YS prepared the illustrations.

## Conflict of Interest Statement

AD is a consultant for A2A Pharmaceuticals (New Jersey) and Salgomed Inc. (San Diego). Y-CS and B-RC declare no conflicts of interest.
